# Nonlinear and machine learning analyses on high-density EEG data of math experts and novices

**DOI:** 10.1038/s41598-023-35032-8

**Published:** 2023-05-17

**Authors:** Hanna Poikonen, Tomasz Zaluska, Xiaying Wang, Michele Magno, Manu Kapur

**Affiliations:** 1 Learning Sciences and Higher Education, ETH Zurich, Clausiusstrasse 59 RZ J2, 8092 Zurich, Switzerland; 2 Integrated Systems Laboratory, ETH Zurich, Zurich, Switzerland

**Keywords:** Cognitive neuroscience, Learning algorithms

## Abstract

Current trend in neurosciences is to use naturalistic stimuli, such as cinema, class-room biology or video gaming, aiming to understand the brain functions during ecologically valid conditions. Naturalistic stimuli recruit complex and overlapping cognitive, emotional and sensory brain processes. Brain oscillations form underlying mechanisms for such processes, and further, these processes can be modified by expertise. Human cortical functions are often analyzed with linear methods despite brain as a biological system is highly nonlinear. This study applies a relatively robust nonlinear method, Higuchi fractal dimension (HFD), to classify cortical functions of math experts and novices when they solve long and complex math demonstrations in an EEG laboratory. Brain imaging data, which is collected over a long time span during naturalistic stimuli, enables the application of data-driven analyses. Therefore, we also explore the neural signature of math expertise with machine learning algorithms. There is a need for novel methodologies in analyzing naturalistic data because formulation of theories of the brain functions in the real world based on reductionist and simplified study designs is both challenging and questionable. Data-driven intelligent approaches may be helpful in developing and testing new theories on complex brain functions. Our results clarify the different neural signature, analyzed by HFD, of math experts and novices during complex math and suggest machine learning as a promising data-driven approach to understand the brain processes in expertise and mathematical cognition.

## Introduction

Current trend in neurosciences is to use naturalistic stimuli which aims to understand the brain functions in the real world during which sensory, cognitive, emotional and motor brain processes overlap^1–4^. Naturalistic stimuli mean complex, dynamic and diverse stimuli which create a more ecologically relevant condition for brain research in comparison to the traditionally used reductionist stimuli^2,4^. Examples of naturalistic stimuli are cinema, classroom biology, video gaming, complex math or listening to a live orchestra^5–9^.

Continuous brain imaging data, which is collected over a long time span during naturalistic stimuli, enables the application of data-driven analyses^2,4^. Machine learning (ML) analyses may assist in generating new hypotheses about the underlying task-relevant brain processes, especially in the naturalistic context. In such contexts, several low and high-level overlapping brain processes occur simultaneously^3^. Due to the overlapping nature of several brain processes, extension of the neuroscientific theories formulated based on reductionist and simplified study designs is both challenging and questionable^2^. Novel methodologies in analyzing naturalistic data are required and data-driven intelligent approaches form a good candidate for developing and testing new theories on the brain functions in the real world^3^.

In addition to the applications for prediction and diagnostics in healthcare^10–14^, ML for brain imaging has application possibilities in the contexts of learning and education^7,2^. For decades, scientists have studied the brain processes during cognitive tasks, like mathematics or language. These studies have brought valuable knowledge on the domain-general brain functions of working memory, attention, and solving strategies (e.g.^15–17^) and domain-specific brain functions on numeric and verbal processing (e.g.^18,19^). Some studies have focused to understand healthy development and expertise^20,21^, whereas others bring insights on disrupted development and learning deficits^22,23^. Neuroscientific studies made in learning sciences have not yet utilized ML in the data analysis. However, ML has potential to be used in data-driven hypothesis formation of the brain functions underlying expertise development or learning deficits, and for real-time adaptive feedback in learning and focused attention^24,25^.

Brain imaging studies with short and simple arithmetic tasks suggest that learning of mathematical knowledge is accompanied by a shift from more frontal to more parietal regions^26–29^. Electroencephalography (EEG) studies suggest that brain processes measured with cortical oscillation and event-related potentials (ERPs) differences are associated with brain functions are modified through expertise, such as including processes related to rote learning and strategy selection for solving the tasks at hand (Hinault and Lemaire for a review^30^). However, a few second simple math tasks, which are used traditionally as stimuli in studies on math expertise, seldom create enough of continuous brain imaging data for which to successfully apply the ML methods. In addition, the commonly used methods in EEG data analysis, cortical oscillation and ERPs, are linear methods which do not capture the nonlinear brain functions.

The brain, like many biological systems, behaves in a nonlinear manner. Nonlinear behavior of biological systems is characterized by a high degree of variability in the time domain (nonstationarity) and randomness that could be attributed to the interaction of internal and external factors influencing the organism^31,32^. Engagement with complex math recruits several cognitive brain processes which overlap with sensory and emotional processes^33,34^. The EEG data collected during such cognitively challenging task is likely highly complex, and therefore, a potentially optimal way to process such data includes an analysis which is suitable for nonlinear systems.

Cognitively challenging tasks create brain states which are clearly different from those of relaxed states^35^. Fractal dimension is a highly sensitive measure in the detection of hidden information contained in physiological time series, has the ability for detection of transients in bio-signals and is shown to vary depending on the brain state^36–38^ functions^39^. An often-used nonlinear measure for signal analysis is Higuchi’s fractal dimension (HFD) which is a measure of signal complexity in the time domain^40,41^. Previous studies utilizing HFD classified successfully different sleep stages and detected the difference in the brain state during drowsiness and wakefulness^42,43^. HFD showed the most robust results and seems to be superior to other FD methods for EEG signals^44,45^.

Comparative studies with linear and nonlinear methods have found a correlation between HFD and alpha power showing an increase in HFD with a reduction of alpha activity^46,47^. Accardo and colleagues^48^ hypothesized that EEG signal can be considered as a fractal curve with decreasing power spectral density following a power law as 1/f (but see also^49^). They suggested that synchronization, corresponding to low signal complexity, could reflect a resting state of cortical networks. On the other hand, desynchronization, corresponding to high complexity, could correspond to active information processing in a certain cortical region^48^. In the comparative studies, several linear methods, including spectral power density, autoregressive model and statistical features in parallel to HFD are studied^43,50^. Radzi and colleagues^51^ showed that the hybrid of fractal dimension, and delta and alpha power have better classifications to the states of arousal than power spectrum alone. S̆us̆máková and Krakovská^43^ compared a huge number of parameters and found that the fractal dimension was the most promising classifier after the fractal exponent significantly discriminating between wake and slow-wave sleep.

A recent study on the disorders of consciousness suggested that differences between lower states of consciousness were 11 times more likely to be detected using HFD than the best performing linear method tested^52^. They also tested machine learning for HFD, reaching an accuracy of 88.6 percent in discriminating among vegetative state, minimally conscious state and healthy controls^52^. In an older study, mental arithmetic task recognition was studied^53^. They reported that the complexity of the EEG signal recorded in the frontal lobe was higher when the subject was performing the mental arithmetic operations than that of the EEG signal recorded when the subject was relaxed. The usage of their HFD spectrum in combination with other features improved the task recognition accuracy in both multi-channel and one-channel subject-dependent algorithms up to 97.87 percent and 84.15 percent, correspondingly^34^. Vega and Noel^54^ also reported HFD as a robust tool for cognitive task discrimination between five states: relaxed state, multiplication, imagining writing a letter, imagining rotation of an object, and erasing and redrawing figures.

This study investigated the neural signature of math expertise with a relatively robust nonlinear analysis, HFD, and explored a new paradigm by applying ML to EEG data collected from math experts and novices when they engaged with long and complex math demonstrations. Our study is the first one aiming to discriminate the cortical functions of math experts from those of novices during long and complex math tasks. Since the pioneering nature of our study, we decided to focus only on one type of features, and based on the previous literature, we chose HFD as the most suitable in distinguishing different cognitive states^43,52,54^. The math demonstrations of this study with a duration of up to 1 min form a part of the current trend in investigating the brain with naturalistic stimuli. In addition, we used the high-density EEG to find the electrodes of interest over which the HFD differs the most and compare the classification accuracy of standard 32-channel electrode distribution to 32 electrodes with the largest HFD difference between experts and novices from the pool of 128 electrodes^53^. Our aim was to describe the EEG data during advanced mathematical cognition with a nonlinear method and evaluate whether the neural signature of math experts and novices differ in a way which is detectable with artificial intelligence. We hypothesized that the experts’ and novices’ brain functions during long math tasks differ in signal complexity over the frontal or parietal regions^18,27,29^ detectable with HFD^43,52,54^, which further, can be classified by a ML model^53,54^.

## Materials and methods

### Participants

Thirty-four math experts (bachelor and master students in math or math-related disciplines, like physics or engineering) and thirty-five math novices (no university-level math studies) participated in the experiment. However, eleven participants from the group of math experts and twelve participants from the novice group were discarded from the data analysis because their EEG data was too noisy, or some of the relevant data was missing due to malfunctioning EEG amplifier. Therefore, in the group of math experts, there were 22 participants (5 female and 17 male), and in the novice group, 22 participants (7 female and 15 male). The background of the participants was screened by a math questionnaire.

The age of the participants ranged from 19 to 24 years (mean 21.0 years) among math experts and from 19 to 35 years (mean 23.8 years) among novices. All participants in both groups were right-handed. No participants reported hearing loss nor history of neurological illnesses. The experiment protocol was conducted in accordance with the Declaration of Helsinki and approved by the Executive Board of ETH Zurich after a review by the ETH Zurich Ethics Commission. All participants provided written informed consent.

### Task design

Participants watched 16 math demonstrations. After each demonstration they were asked three self-evaluation reflections to which they answered by pressing a button in a 4-button response box. Each set of trials consisted of four excerpts of the same presentation style (symbolic or geometric), and these sets were presented in a pseudo random order via a monitor. The pseudo randomization defined the presentation order (symbolic first or geometric first). However, each participant saw the same four math demonstrations presented in both symbolic and geometric form before seeing them in the other form.

Each math demonstration consisted of several slides, from 4 up to 12 slides (6.9 slides on average) depending on the complexity of each demonstration. The total duration of math demonstrations varied from 13 to 68 s (33.1 s on average). The timing of each slide was the same for all the participants. The duration of each slide was defined according to an online screening in which 25 math experts and 25 math novices watched the math demonstrations slides and auto-regulated the following slide with a button press. The participants who attended the online screening did not attend the actual EEG experiment. The duration of each slide in the EEG experiment was the average time the participants spent on each slide during the online screening. In the online screening, there was no statistically significant difference between experts and novices in the duration of time spent on each slide.

### Data acquisition

The stimuli were presented to the participants with the MATLAB via PsychToolbox. The experimenter launched the playback of the presentation program after which participant could navigate to the math demonstrations by a button press once they had read the instruction slides on the screen. The total length of the experiment material was approximately 15 min.

The data were recorded using Ant Neuro eego mylab electrode caps with active 128 EEG channels (https://www.ant-neuro.com/products/eego_mylab).

Four external electrodes placed below, above and on the left side of the left eye and on the right side of the right eye. The offsets of the active electrodes were kept below 30 mv at the beginning of the measurement, and the data were collected with a sampling rate of 2048 Hz. A timestamp (trigger) was marked into to EEG data at the beginning of each slide of the math presentations. The triggers were sent wirelessly via Lab Streaming Layer (https://github.com/sccn/labstreaminglayer).

### Data pre-processing

The EEG data of all the participants were first preprocessed with EEGLAB (version 2019.1^55^). The reference was set as the average of all the EEG electrodes. The data were high-pass filtered at 0.5 Hz and low-pass filtered at 40 Hz. In preprocessing, we used high-pass filtering over 0.5 Hz because it is a standard procedure and shown to improve the data quality the most^56^. The frequency bands over 40 Hz were filtered out because of the 50 Hz line noise. It is a common procedure to use a wide frequency spectrum for HFD analysis. High-pass filtering varying between 0.1 and 2 Hz and low-pass filtering between 30 and 70 Hz for HFD is used in previous studies^39,46,47,51–53,57–59^.

Finite impulse response (FIR) filtering, based on the firls (least square fitting of FIR coefficients) MATLAB function, was used as a filter for all the data. Then, the data were treated with independent component analysis (ICA) decomposition with the runica algorithm of EEGLAB^55^ to detect and remove artefacts related to eye movements and blinks. ICA decomposition gives as many spatial signal source components as there are channels in the EEG data. Typically, one to four ICA components related to the eye artefacts were removed. Noisy EEG data channels for some participants were interpolated.

### Feature extraction

#### Higuchi fractial dimension (HFD)

The EEG time-series has a duration between 10 and 20 min, resulting in a large data size per sample. Hence, feature extraction is necessary to capture relevant information. The extracted features are then used to draw conclusions regarding the relevance of each brain area for mathematical calculations. For this purpose, the fractal dimension (FD)^60^ for each sample is calculated and is used to measure the complexity of the signal. A simple pattern that is repeating continuously can become a very complex series which is the basis for the fractal constructs. A fractal is a shape that retains its structural detail despite scaling and is the reason why complex objects can be described with the help of fractal dimension. One variant of FD, the Higuchi’s fractal dimension^40^, has its roots in chaos theory and has been successfully applied as a complexity in various domains of signal processing. It has been shown to be a good numerical solution to nonlinear signals^61^. The speed, accuracy, and cost of applying the HFD method for research and medical diagnosis make it stand out from the widely used linear methods^57^. Among the different FD algorithms, Higuchi’s method^61^ demonstrates to be a more accurate option for EEG signals, since it is accurate for stationary and non-stationary signals.

Say $\textbf{X}$ is an EEG signal of length *T* and N is the length of a time window on which we calculate a HFD value. A new signal $x_m^{k}$ is constructed from $\textbf{X}$, with window size N where m = (1, 2, ..., k) denotes the starting point and k = (1, 2, ..., $k_{max}$ ) the interval size:

1 $$\begin{aligned} x_m^{k} = \left\{ x(m), x(m+k), x(m+2k), ... , x\left( m+ \Bigl \lfloor \frac{ N - m}{ k } \Bigr \rfloor \right) \right\} \end{aligned}$$

$L_m(k)$ describes the length of the curve of $x_m^{k}$ for every k given m:

2 $$\begin{aligned} L_m(k) = \frac{\sum _{i=1}x(m+ik)-x(m+(i-1)k)(N-1)}{\Bigl \lfloor \frac{ N - m}{ k } \Bigr \rfloor k} \end{aligned}$$

where $\frac{N-1}{\Bigl \lfloor \frac{ N - m}{ k } \Bigr \rfloor }$ is the normalization factor. Length *L*(*k*) is defined by the average of the k lengths:

3 $$\begin{aligned} L(k) = \frac{1}{k}\sum _{m=1}^{k}L_m(k) \end{aligned}$$

HFD is the slope of the best fitted curve between all the data points of time-series X for a given time window N for for k = (1, 2, ..., $k_{max}$ ) between log(1/k) and log L(k):

4 $$\begin{aligned} HFD(N,k_{max}): \text {best fit of}\ \left\{ \left( log\left( \frac{1}{k}\right) \right) , log(k) \right\} \end{aligned}$$

It is possible to calculate HFD for the whole signal $(T=N)$. However, this is not recommended if the signal is nonstationary. In such cases the HFD value does not represent the true measure, and division into windows (or segments) is advised. In^48^, Accardo and colleagues have shown on synthetic fractal signals that Higuchi’s algorithm is more efficient, faster, more accurate and able to estimate fractal dimension for short segments, compared to Maragos and Sun’s algorithm proposed in^62^.

#### Hyperparameter tuning

An important hyperparameter that requires finetuning is $k_{max}$. There is no agreed methodology to optimize this parameter^63^. As per Eq. (3), HFD is summed up to $k_{max}$, therefore increasing $k_{max}$ will lead to an increase in HFD. A poor choice of $k_{max}$ will result in uninformative HFD, thus, it has to be carefully tuned.

We propose the following methodology to identify the best value for $k_{max}$:

1. We compute the HFD values as per Eq. (4) for a wide range of $k_{max}$ values, i.e., $k_{max} \in {2, 5, 20, 100, 150, 200, 400}$ over all subjects and presentations.
2. We identify the $k_{max}$ at which the difference (Eq. 5) between HFD values of significant and non-significant channels is maximized. Significance/non-significance is assessed by taking the maximum/minimum HFD value across all electrodes for a subject. Here, the minimum value is understood as the baseline fractal dimension and is therefore subtracted from the maximum value, which is the complexity of the relevant channels. We base this requirement on the assumption that certain EEG regions are more relevant than others for the mathematical tasks. Hence, there will be a difference in HFD values and we want to select the $k_{max}$ that maximizes this difference.
3. The $k_{max}$ value that satisfies the above requirement is chosen to compute the HFD values for further analyses and for the machine learning classification.

#### HFD features analyses

Estimating HFD values for each channel of each participant allows to investigate which brain areas are most active while performing mathematical tasks. Since HFD values have no physical interpretation, a relative comparison between two different groups is performed.

First, a comparison between experts and novices is investigated, by taking the average of all HFD values of the expert group and the novice group and subtracting them from each other:

5 $$\begin{aligned} \Delta HFD_{ch_i} = \overline{\overline{HFD_{expert_j, pres_k}}}_{ ch_i} - \overline{\overline{HFD_{novice_j, pres_k}}}_{ch_i}, \end{aligned}$$

where $j\in${1,...11} is the index of experts and novices, respectively, $k\in${1,...16} is the index of presentations and $i\in${1,...129} is the index of EEG channels.

A one-sided t-test is calculated, testing whether there is a significant difference between the two groups. A visual heatmap of the difference between experts and novices based on Eq. (5) is mapped onto the head for better qualitative interpretation.

Subsequently a more fine grained analysis is performed by comparing the difference between expert and novice for algebraic and geometric separately:

6 $$\begin{aligned} \Delta _{AG}HFD_{ch_i} = \overline{\overline{HFD_{expert_j, pres_{k_A}}}}_ {ch_i} - \overline{\overline{HFD_{expert_j, pres_{k_G}}}}_{ch_i}, \end{aligned}$$

where $k_A$ and $k_G$ $\in${1,...8} is the index of the algebraic and geometric presentations respectively.

#### Machine learning classification

We posit the question if a prediction can be made whether a new subject is a novice or an expert based on EEG recordings while performing mathematical tasks. We frame this problem as a two-class classification task. To understand and interpret the outcome of the machine learning classifiers, care needs to be taken while generating the classification dataset and splitting it into training and testing sets.

We first define the classification-dataset as a collection of subject-presentation pairs (e.g. Expert1-Presentation1A etc.). Together with the 16 presentations, the full dataset include 704 samples, i.e., subject-presentation pairs. Subsequently, we calculate either a unique HFD value per EEG channel, meaning that each sample consists of 124 HFD features, or divide the EEG signals of total length T into non-overlapping windows of length N and calculate a HFD value for each window leading to (T/N)*124 HFD features. To be noted that the channels “VEOGL”, “HEOGL”, “HEOGR”, “VEOGU”, “HEART” are discarded, since they do not record brain signals but eye movement and cardiac activity.

**Figure 1:**
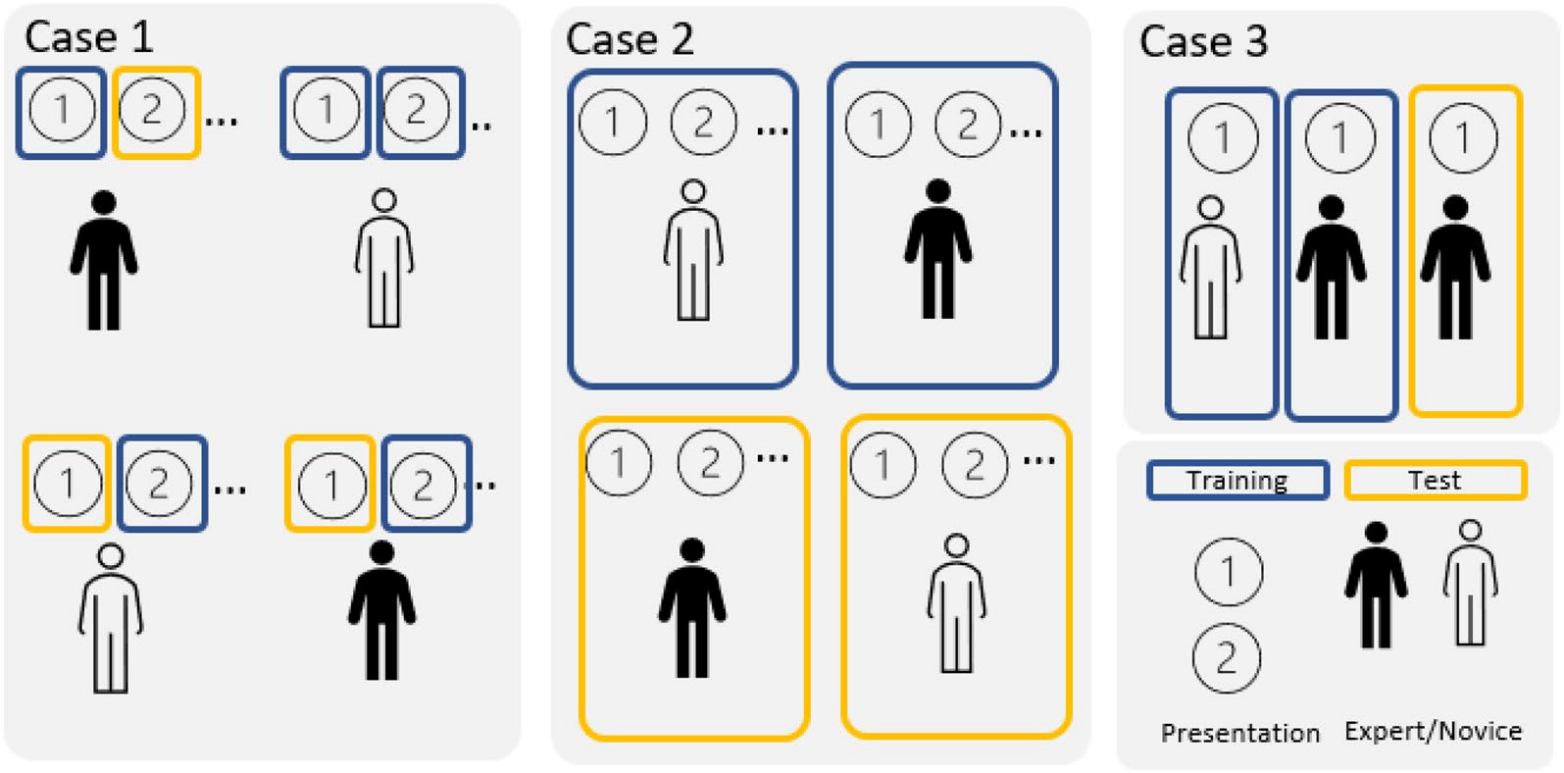
Classification-dataset split illustration. Case 1: Subject-presentation pairs split, Case 2: Subject-specific split, Case 3: Presentation-specific split.

Since this work is the first in the literature to attempt an automatic classification of mathematical cognitive behavior, we propose three different cases of dataset splitting, illustrated in Fig. 1:

1. Subject-presentation pairs: We randomly split all 704 samples without considering whether a sample is coming from different subjects. This means that the samples from the same subject can either be entirely in the training set or in the validation set, or partially in the training and in the validation set.
2. Subject-specific: We split the dataset on the level of subjects, meaning that all subject-presentation pairs of the same subject are either in the training or validation set.
3. Presentation-specific: We deal with each presentation as a separate machine learning task. In other words, we divide the full dataset into sub-datasets, each of which consists in a single presentation, and perform the training and testing procedure on each of the sub-datasets.

With case 1, we verify if the machine learning (ML) classifier is able to discern between the 22 experts and novices present in the dataset based on a single mathematical presentation. With case 2, we validate the ML classifier on new subjects of which data it has never seen before. The former is a relatively easier classification task, but necessary as a first proof-of-concept, whereas the latter tackles the most challenging problem of inter-subject variability common to all biomedical data. With case 3, we analyze whether a prediction can be made based on samples coming from a single presentation. By training a separate classifier for each presentation, we can compare the classification accuracy among the presentations and draw insights about which mathematical presentation is more suitable for discerning between math novices and experts.

For cases 1 and 2 we calculate a single HFD value per EEG channel throughout the whole duration of the presentations. This choice is motivated by the fact that all presentations, of different recording lengths, belong to the same dataset on which a machine learning classifier is trained on and, in general, the classifiers require a fixed numbers of features. This is no longer an issue for case 3, because each sub-dataset consists of data from a single presentation of fixed length. Hence, we can increase the granularity and use a non-overlapping moving window of length N to calculate the HFD value in Eq. (4) for each window. More precisely, a HFD value is calculated every N seconds of the duration of the presentation $HFD_{1:N},..., HFD_{t:t+N}$ with *t* being time steps. This allows to analyze the temporal evolution of the presentation and draw conclusions regarding the classification differences. We test several values of N, i.e., 5, 8, 11 s.

Once the datasets are prepared, we proceed with classifiers training using the scikit-learn Python package. We investigate several ML algorithms including Nearest Neigbours, Linear SVM, Decision Tree and Adaboost. We first optimize the classifiers by tuning the hyperparameters under case 1, i.e., subject-presentation level. Once the optimal parameters are found, we keep them for case 2 and 3.

**Table 1:** Machine Learning algorithms used for classification between experts and novices.

Algorithm	Parameters
Nearest Neighbors	Number neighbors: 3, 5, 7, 9, 11
Linear SVM	Kernel: linear, C: 0.025, 0.5, 0.75
Decision Tree	Maximum depth: 3, 5, 7
AdaBoost	Number estimators: 25, 50, 100

The various ML algorithm tested are summarized in Table 1, with their corresponding parameters ranges. Once the best performing ML algorithm has been identified, we further optimize it with a grid-search algorithm. Given the small sample size, ten fold cross-validation (90 percent training/ 10 percent validation set) has been applied with a fixed seed.

## Results

**Figure 2:**
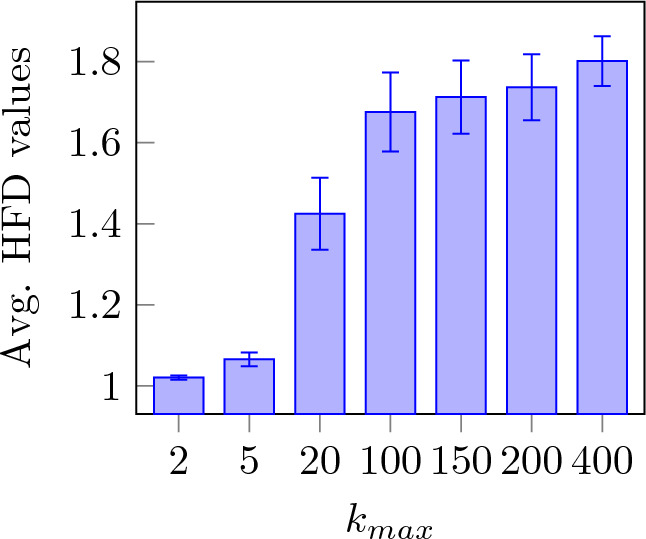
HFD value, averaged across all channels all subjects and presentations for different values of $k_{max}$.

**Figure 3:**
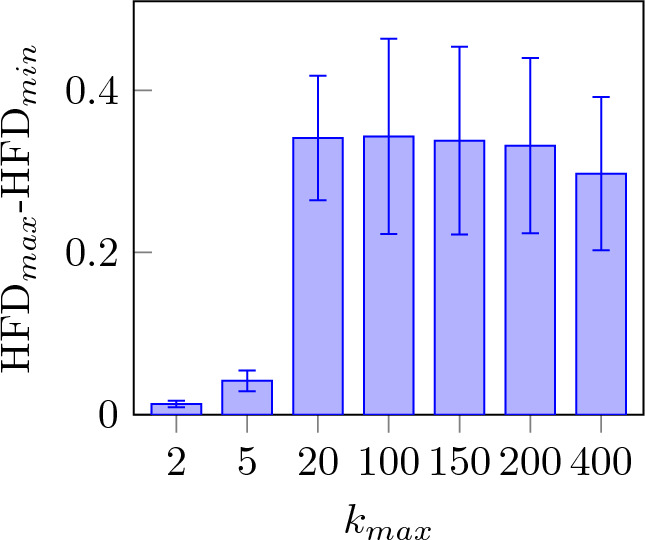
HFD value, difference between HFD values between the maximum and minimum of all channels averaged across all subject and presentations for different values of $k_{max}$.

**Figure 4:**
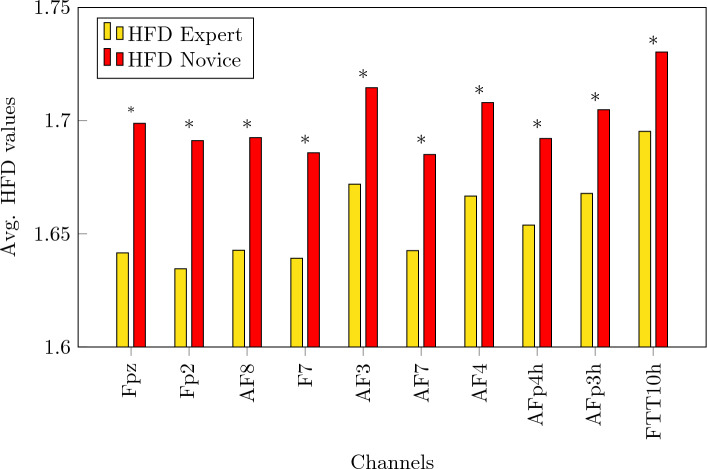
Top 10 channels with the highest difference between their HFD values. Asterisk $*$ indicates that the average value of HFD expert is statistically different than HFD novice under *p* = 0.05 threshold for that specific channel.

As described in the introduction, extracting the neural signature of math experts and novices requires careful features extraction via the HFD method. To calculate the HFD correctly, hyperparameter $k_{max}$ requires finetuning. Therefore, “Optimal *k*_max_” section presents the optimization results of hyperparameter $k_{max}$. Based on the extracted HFD features, experts and novices are compared in “HFD feature analyses” section giving insights which brain region is relevant for performing mathematical tasks. Finally, based on the features, classification results between experts and novices are shown in “Expert/Novice classification” section.

**Figure 5:**
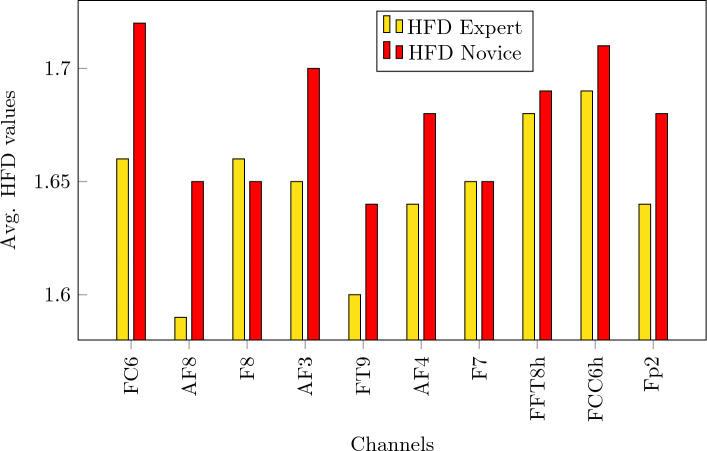
Top 10 channels with the highest difference between their HFD values of the resting state. None of the average values of HFD experts is statistically different than HFD novice under *p* = 0.05 threshold for that specific channel.

### Optimal $k_{max}$

Figure 2 shows the value of HFD for all subjects averaging over all channels for different values of $k_{max}$. HFD is steadily increasing but starts to plateau at a value of 100. Figure 3 shows the difference between the maximum and minimum HFD values for different $k_{max}$ with accordance to Eq. (5). It can be observed that the difference in HFD value corresponding to $k_{max}$ reaches a peak at 20 and 100 and progressively declines with increasing $k_{max}$. Based on the fact that HFD is plateauing at $k_{max}$ equal to 100 and the largest difference between the maximum and minimum HFD values is also found at the same value, $k_{max}=100$ is used for further analysis.

### HFD feature analyses

**Figure 6:**
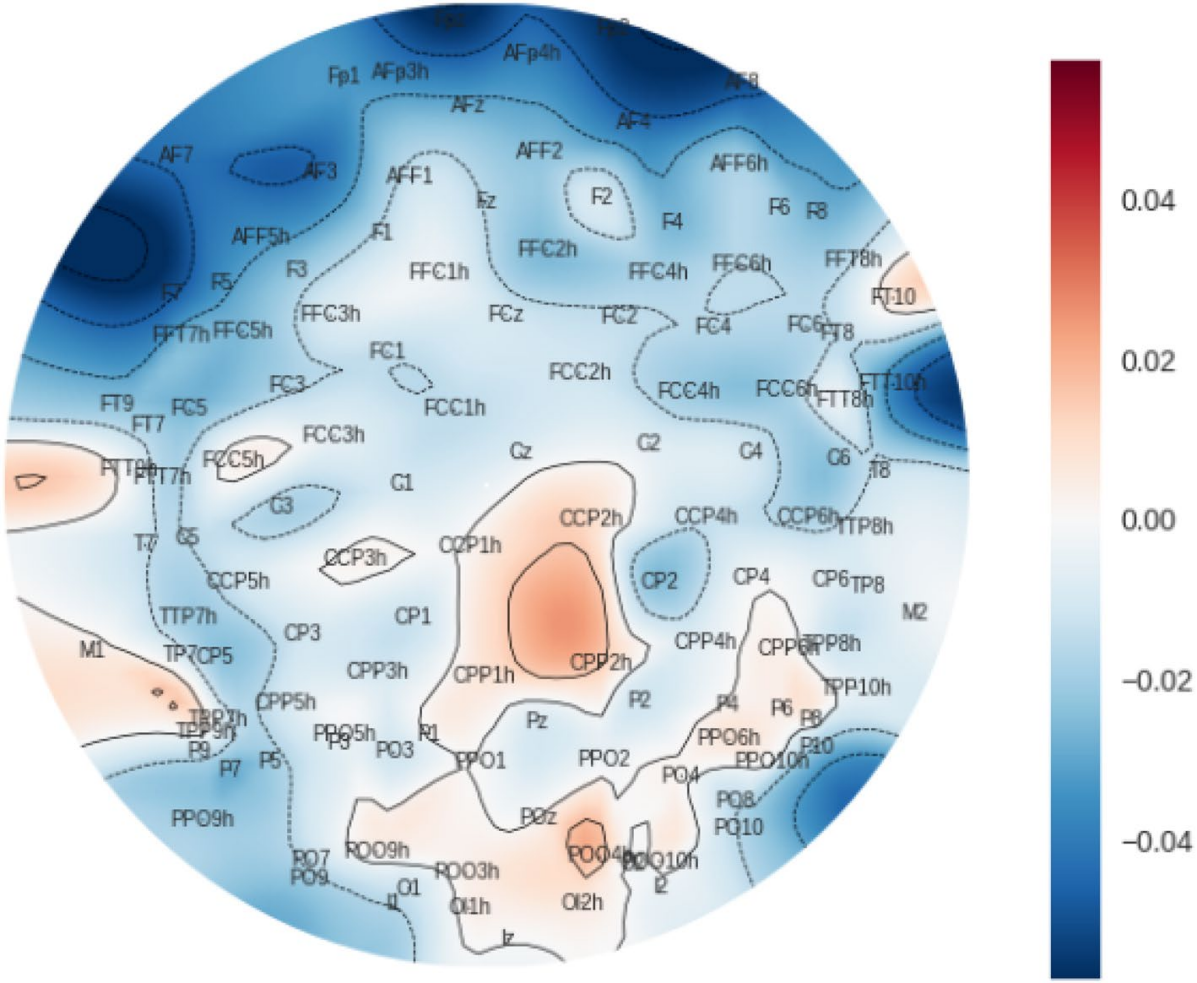
Heatmap of HFD difference between Expert and Novices, generated using MNE-Python package^64^. The darker blue and red colors respectively indicate the brain areas where the positive and negative differences between experts and novices are the largest.

Figure 4 shows the difference between the average HFD values between experts and novices, for the top 10 channels that present the highest difference between expert and novices. All top 10 channels are statistically significant under *p* = 0.05 constraint. All channels are depicted in form of a heatmap in Fig. 6. The dark blue shaded areas indicate the highest positive difference between expert and novices.

To evaluate if these differences are pre-existing, independently from being a math novice or math expert, we calculate and compare the HFD values from the resting state EEG data with eyes open of the two groups of subjects. Figure 5 shows the HFD values of the channels with highest difference between experts and novices in resting state, where the subject do not perform any cognitive task. There is no statistically significant difference between experts and novices in this case. It suggests that the math presentations given as stimuli are effective in evoking different brain activations and that the HFD features are a valid method to extract such differences between the two groups.

**Figure 7:**
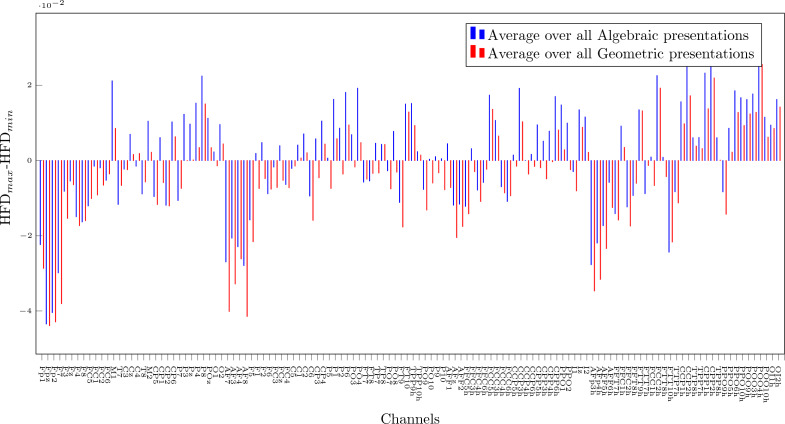
HFD$_{max}$-HFD$_{min}$ calculated as average for all Algebraic versus Geometric presentations for all channels as defined in Eq. (6).

The subsequent more finegrained analysis comparing the difference between expert and novice for algebraic and geometric is shown in Fig. 7 given Eq. (6). Although there are differences between algebraic and geometric presentations, none of them is statistically different under *p*-value 0.05 hypothesis.

### Expert/Novice classification

Table 2 summarizes the classification results between expert and novices. With the subject-presentation split, the accuracy reaches 97% demonstrating that it is possible to automatically classify between math experts and math novices based on their electroencephalogram (EEG) signals while watching math demonstrations because the ML model can successfully learn each subject’s brainwaves signatures.

**Table 2:** Classification results between experts and novices based on different classification algorithms for Subject-presentation pairs, Subject-specific and Presentation-specific split. All results are based on ten-fold cross validation and averaged over 3 random seeds. The classification results using 32 channels in the standard 10/20 system are reported in parenthesis.

	Subject-pres. pairs	Subject specific	Presentation specific split													
			1A	1G	2A	2G	3A	3G	4A	4G	5A	5G	7A	7G	9A	9G
Nearest Neighbors	97% (94%)	48%	48%	61%	52%	51%	49%	45%	60%	47%	53%	66%	67%	57%	43%	55%
Linear SVM	87% (63%)	66%	56%	53%	53%	54%	46%	61%	49%	43%	63%	46%	63%	49%	39%	61%
Decision Tree	78% (66%)	50%	49%	47%	55%	59%	52%	53%	51%	41%	55%	50%	79%	49%	47%	42%
AdaBoost	93% (84%)	56%	53%	51%	45%	51%	49%	59%	52%	39%	66%	59%	61%	39%	47%	56%
**Best**	**97%**	**66%**	**56%**	**61%**	**55%**	**59%**	**52%**	**61%**	**60%**	**47%**	**66%**	**66%**	**79%**	**57%**	**47%**	**61%**

The best accuracy values are in bold.

However, when we split the training and test sets on a subject level, meaning that we increase the difficulty of the task by introducing inter-subject variability that is well-known to be challenging in biosignals classification, i.e., the trained model is validated on new subjects whose data it has never seen before, the accuracy falls to 66%.

So far the results are shown by considering all presentations for each subject, i.e., the calculated HFD features for all presentations are concatenated for the final classification stage. We suspect that the poor classification accuracy could be partially caused by some of the presentations that might perform poorly. Hence, we perform presentation-specific classification on subject level and the classification accuracy improves up to 79% (presentation 7A).

**Figure 8:**
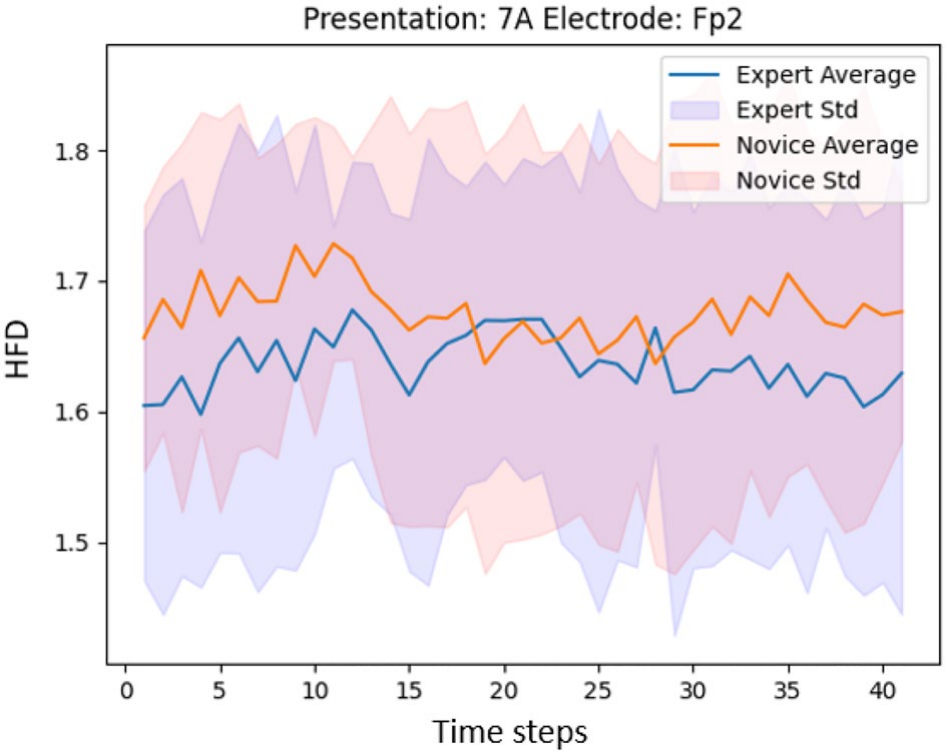
HFD values (before averaging) for presentation 7A, channel FP2 for Experts (average) and Novices (average).

**Figure 9:**
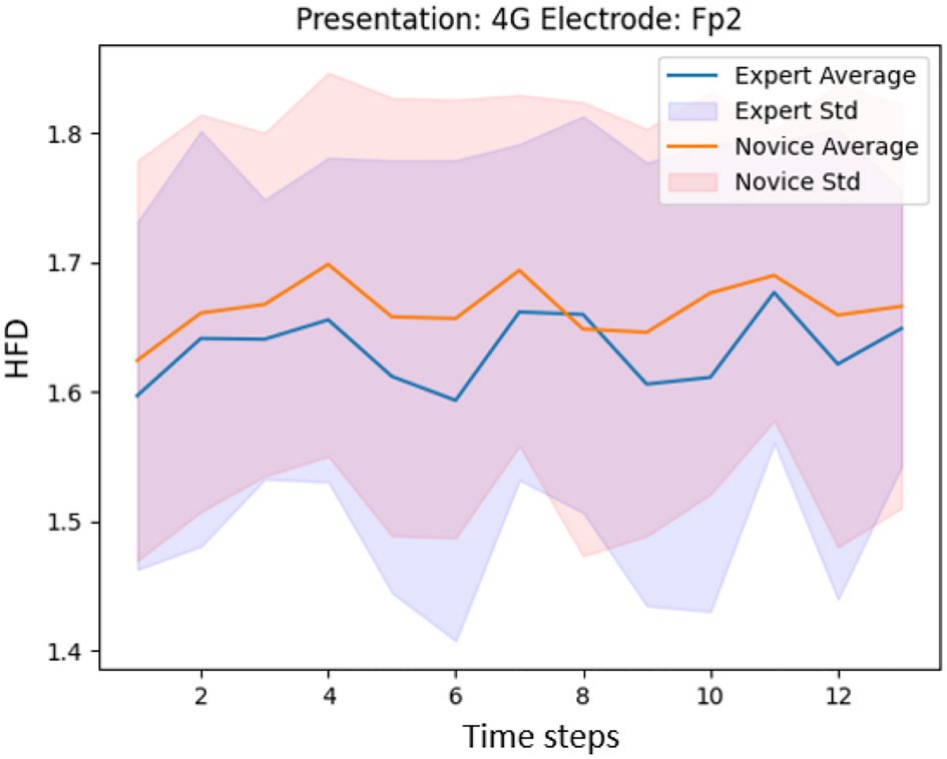
HFD values (before averaging) for presentation 4G, channel FP2 for Experts (average) and Novices (average).

Figures 8 and 9 show the HFD values when window size of 8 s is applied for the presentation with the highest (presentation 7A) and the lowest (presentation 4G) classification accuracy. The difference in classification accuracy may be explained through a better separation between Experts and Novices in HFD features.

We further analyze if it is necessary to have high-density EEG data for the classification. We reduce the number of EEG channels from 124 to 32 according to the international 10/20 system. The results, reported in Table 2, demonstrate that reducing the number of channels yields decreased classification accuracy. Moreover, the channels with the highest difference in HFD values between the two groups, shown in Fig. 4 in brackets, are absent in the 32-channel standard configuration. Hence, as a pilot study, the usage of a high-density EEG setup has proven to be beneficial. In future work, we recommend to investigate the possibility to reduce the number of channels as it yields less obtrusiveness and more comfort for the participants.

## Discussion

Advantages of ML for brain research include the data driven approach which enables generation of hypotheses about underlying brain processes in rest or in active engagement with a cognitive or emotional task. Such underlying processes are sometimes impossible to detect by experts’ observations. ML also enables explorations of new paradigms with respect to their neurophysiological signatures. One of such new paradigms is naturalistic study design which aims to understand the brain during real-life tasks, like when solving complex math.

Our novel approach on applying ML to EEG data recorded in math experts and novices during complex math encourages to expand the usage of data driven brain imaging methods from healthcare to education. Our approach utilizing nonlinear HFD, which measures signal complexity, was reliable in describing the data by systematically detecting the difference in the neural signature of math experts and novices with a 98% cross-validation accuracy. However, the results gained with ML discriminative algorithm were mixed and showed 50–80 percent classification accuracy when tested with unseen subjects.

Nonlinear fractal dimension methods seem ideal for tracing fluctuations in biological systems, including the brain, which are nonlinear by nature. HFD is a measure of signal complexity in the time domain^40,41^ and has been successfully applied for brain state analysis of EEG in sleep, drowsiness, wakefulness and different cognitive states^37,42,53,54^. Our results gained with HFD show a difference in the neural signature between math experts and novices during long and complex math tasks with a high classification accuracy. These results encourage to use the HFD method in detecting subtle differences in the brain states, like those of math experts and novices, which go beyond the more drastic differences in the brain states during the levels of arousal, like sleep stages, or drowsiness and wakefulness.

Despite the successful classification to experts and novices based on HFD was relatively stable for the entire dataset, the ML model adapted poorly to unseen subjects, and we could not overcome the overfitting and high generalization error caused by inter-subject variability. The most important reason for such a poor generalization is that our dataset is incorrigibly small to be divided into the training and test sets on a subject level. In healthcare, big data platforms are being formed increasingly (Eickhoff et al., 2016; Zbontar et al., 2019), and it is important to take similar steps to create large and clearly labeled open data pools for educational neurosciences.

Our small dataset may function reasonably well for method development of data-driven approaches, since the differences between math demonstrations are statistically significant especially over several frontal electrodes showing higher frontal signal complexity in math novices in comparison to experts. Cognitively, these results may indicate novices’ stronger recruitment of domain-general processes in comparison to experts, which is in line with previous literature^18,17^.

Some studies have investigated the connection between nonlinear FD methods and linear oscillation analyses over delta, theta and alpha bands. These studies show a dependence between the nonlinear and linear methods and suggest that the most reliable results are gained when combining nonlinear and linear methods to classify different brain states^18,43,51^, (Acharya et al., 2005). Since combination of nonlinear and linear methods seem to bring the most robust classification results, we could combine the HFD and oscillation analyses and feed the combined information to a machine learning model. Our novel analysis with machine learning utilized only fractal dimension; however, we report on other papers the brain oscillations for the same dataset (Formaz et al., unpublished data^9^.

Another interesting way to deepen the analysis of our dataset was to break the temporal data stream to segments. With a larger dataset and statistical power, time points during which the neural signatures of math experts and novices differ the most could potentially be found. This data-driven approach may have practical implications after detecting whether the cortical functions of experts and novices differ the most at the beginning, at the end, or at some other time point during the math demonstrations. With our dataset, ML algorithm showed 50–80 percent classification accuracy for unseen subjects when breaking the data to a temporal stream. Such a high variation may be explained by a small dataset, or by a combination of several features related to the length, content, and difficulty level of the math demonstrations.

Understanding which parts of the math demonstrations to emphasize when teaching complex math may be helpful in supporting students’ development towards math expertise. Such time-dependent information may be hard to collect with questionnaires or other behavioral measures, and therefore, brain-originated data-driven methods may be the only way to access such information in the context of learning. Further, these ML models could be used to create learning contexts in which adaptive feedback is given to adjust to the individual needs of a learner or those of a specific group during collaborative learning, building on the previous examples like BCI applications for post-stroke motor rehabilitation, or relatively simple neurofeedback applications for focused attention or working memory^11,24,25,53^. Simple options for BCI interventions for the math demonstrations used in our study might be to adjust the velocity of presenting new information, or by scaffolding the learning process via instructions or remarks depending on the EEG signal of the learner.

## Limitations

Our novel paradigm combining mathematical cognition, cortical activity and ML is exploratory in nature and we recognize the following limitations. First, the most drastic limitation is the small dataset in use. The straightforward way around it would be to increase significantly the amount of data, e.g., by at least doubling the number of participants. The more data the better we can estimate the real data distribution of the general population. The second limitation is related to the classes chosen for the ML classification. We chose to compare two groups of participants during the same cognitive task. Other strategy for a small dataset would be to explore individual differences, for example, by aiming to classify the data excerpts of resting state and cognitively active state for each participant. Earlier studies show that differentiation of brain states for an individual participant during simple sensory tasks is rather robust whereas the generalizations of the cortical activation patterns across a group of participants, and during complex cognitive tasks, is challenging. However, such individual brain state classification would not give us hardly any insights for the expert-novice differences during mathematical cognition. As the third limitation to consider, when preprocessing, we chose to band-pass filter the data with a bandwidth of 0.5–40 Hz due to the contamination of the data with the 50 Hz line noise. HFD is associated with changes in delta, theta and alpha oscillations which all were included in our analysis. However, also gamma oscillation is known to be important during cognitive tasks, and it has been connected to HFD. Due to bandpass filtering chosen, gamma activity is not included in our analysis. Based on previous literature, HFD seems the most stable fractal dimension methods^61^. However, as the fourth limitation of our study, is the general criticism for the HFD that it has a short margin of scale which may give the same complexity number to signals with only subtle differences. For detecting the possibly small differences in the cortical activity of math experts and novices, some other method with more detailed scale may be more suitable. Fifth, for the cross-validation, different models could be compared to find a model with ideal complexity which balances between overfitting of an unnecessarily complex model and simple model’s inability to adapt to the details of the complex cognitive data. Ideally for ML algorithms, each sample (e.g. EEG data collected during each math demonstration) would have the same number of data points (e.g. the same duration). However, it is difficult to realize in practice due to different duration it takes to solve different naturalistic math tasks. In the future, research of brain processes during abstract cognition might be conducted, for example, within a video game context, in which the duration is easier to match to be the same over all the rounds played. The sixth limitation is in our study design, in which we did not have any cognitive task different to mathematics which makes it difficult to evaluate whether the differences in HFD between math experts and novices were related to the math tasks per se, or if we had noticed the same difference with any cognitive task, for example related to history or language. However, a previous study comparing math experts and novices, showed that the brain activation differed only during math tasks but not during other cognitive tasks on the same difficulty level^18^.

## Conclusions

The present study used a unique paradigm to compare neural correlates of math experts and novices while solving naturalistic math demonstrations. Overcoming limitations of previous studies with reductionist stimuli and linear EEG analysis methods, the brain functions during abstract cognition were measured with a high-density EEG during long and complex math demonstrations and analyzed with a relatively rigor nonlinear method, HFD. Our results indicated that math novices have a higher signal complexity measure with HFD than experts over several frontal electrodes suggesting a stronger engagement of domain-general brain functions. Further, we explored ML algorithms for classifying math experts and novices based on their neural signature. These results were promising but we also acknowledge the inevitably small dataset we had in use for consistent results. We encourage taking example from brain imaging databases created in healthcare for a creation of a similar database for educational neuroscience. In the future, application possibilities for such a database and deep learning lay in data-driven theory formation for normal and disrupted learning and development, and adaptive feedback systems for learning contexts.

## Data Availability

The data will be openly published in autumn 2023, and until then, the data can be received by requesting them from the corresponding author.
